# Digital Informed Consent/Assent in Clinical Trials Among Pregnant Women, Minors, and Adults: Multicountry Cross-Sectional Evaluation of Comprehension and Satisfaction

**DOI:** 10.2196/65569

**Published:** 2025-08-15

**Authors:** Jaime Fons-Martinez, Carlos Murciano-Gamborino, Cristina Ferrer-Albero, Carlos Vergara-Hernandez, Javier Diez-Domingo

**Affiliations:** 1 Vaccines Research Department, FISABIO (Foundation for the Promotion of Health and Biomedical Research of Valencia Region), Avenida Cataluña, 21, Valencia, 46020, Spain, 34 961925948; 2 CIBER de Epidemiología y Salud Pública, Instituto de Salud Carlos III, Madrid, Spain; 3 Universidad Católica de Valencia San Vicente Mártir. Facultad de Medicina y Ciencias de la Salud, Valencia, Spain; 4 Statistics Service, Platforms and Services, FISABIO (Foundation for the Promotion of Health and Biomedical Research of Valencia Region), Valencia, Spain

**Keywords:** informed consent, eConsent, clinical trials, comprehension, pregnant women, adults, participant satisfaction, demographic factors, eHealth, electronic consent materials, pregnant, women, minor, youth, adolescent, teenager, older adult, electronic informed consent, digital informed consent, statistical analysis

## Abstract

**Background:**

Informed consent (IC) is a cornerstone of ethical clinical research, yet comprehension gaps persist. The i-CONSENT guidelines aim to improve IC materials by enhancing clarity, accessibility, and tailoring them to the needs of diverse populations. This study evaluates the effectiveness of electronic IC (eIC) materials developed under these guidelines for 3 target populations—minors, pregnant women, and adults—across Spain, the United Kingdom, and Romania.

**Objective:**

The primary aim of this study is to assess participants’ comprehension of and satisfaction with eIC materials tailored to their specific needs. The secondary objectives are to identify demographic predictors of comprehension, evaluate the cross-cultural applicability of materials, and explore format preferences.

**Methods:**

A cross-sectional study was conducted with 1757 participants (620 minors, 312 pregnant women, and 825 adults), who reviewed eIC materials through a digital platform offering layered web content, narrative videos, printable documents, and infographics. Materials were co-designed using participatory methods, including design thinking sessions with minors and pregnant women, and online surveys with adults. Comprehension was assessed using an adapted version of the Quality of the Informed Consent questionnaire. Objective comprehension (part A) was categorized as low (<70%), moderate (70%‐80%), adequate (80%‐90%), or high (≥90%). Subjective comprehension (part B) was measured using a 5-point Likert scale. Satisfaction was evaluated through Likert scales and usability questions, with scores ≥80% considered acceptable. Multivariable regression models were applied to identify predictors of comprehension.

**Results:**

Objective comprehension exceeded 80% across all groups: minors (mean 83.3, SD 13.5), pregnant women (mean 82.2, SD 11.0), and adults (mean 84.8, SD 10.8). Women/girls outperformed men/boys (β=+.16 to +.36). Generation X adults scored higher than millennials (β=+.26, *P*<.001), while prior trial participation was associated with lower comprehension scores (β=−.47 to −1.77). Among minors, compared with participants from Spain with no previous clinical trial experience, comprehension was significantly lower in Spain (*P*=.03), Romania (*P*<.001), and the United Kingdom (*P*<.001). Format preferences varied: 382 out of 620 (61.6%) minors and 152 out of 312 (48.7%) pregnant women preferred videos, whereas 452 out of 825 (54.8%) adults favored text (*P*<.001). Satisfaction rates surpassed 90% in all groups (minors, 604/620, 97.4%; pregnant women, 303/312, 97.1%; and adults, 804/825, 97.5%), with 777 out of 825 (94.2%) adults also indicating that the materials facilitated understanding. While translated materials maintained high efficacy across countries, comprehension scores in Romania were lower among participants with lower educational levels (β=−1.05, *P*=.001). Materials cocreated in Spain were effective across countries but yielded higher comprehension within the original target population.

**Conclusions:**

eIC materials developed following the i-CONSENT guidelines achieved high levels of comprehension and satisfaction across diverse populations, demonstrating scalability for multinational trials. Cocreation and multimodal design effectively addressed participant preferences; however, cultural adaptation remained crucial for optimizing outcomes. The negative impact of prior trial participation highlights the need for tailored engagement strategies for returning participants. Future research should explore regional disparities, evaluate interventions for overconfident returning participants, and validate these tools across broader cultural contexts.

## Introduction

### Background

Informed consent (IC) is a key element in ensuring the autonomy of potential participants when deciding whether to partake in a study. To be effective, IC must meet 5 key criteria: voluntariness, capacity, disclosure, understanding, and decision-making. However, numerous studies have highlighted persistent gaps in participants’ comprehension of the information provided during the IC process [1].

The i-CONSENT project was initiated to address these challenges by improving IC materials to make them more comprehensible, accessible, and tailored to the specific needs of diverse populations. As part of this initiative, the project developed the “Guidelines for Tailoring the Informed Consent Process in Clinical Studies” [2] (i-CONSENT guidelines). Among the recommendations included in the guidelines, involving potential participants in the preparation of the IC and its associated materials has been identified as a key factor [3].

To evaluate the impact of these guidelines, 3 mock studies were conducted, each focusing on a distinct population: minors, pregnant women, and adults. The IC materials were presented in electronic formats (electronic IC [eIC]), incorporating innovative features such as layered web content, narrative videos, printable documents, and customized infographics. These formats allowed participants to engage with the information in ways that suited their preferences and cognitive styles.

This study aims to assess participants’ comprehension of and satisfaction with eIC materials tailored to their specific needs. Additionally, it seeks to explore cross-cultural applicability by implementing these materials across 3 countries—Spain, the United Kingdom, and Romania—and examining demographic predictors of comprehension. The findings contribute to advancing inclusive and participant-centered IC processes in clinical research while promoting informed decision-making.

### Objective

The main objective of this study is to estimate the proportion of potential participants who understand the 3 different eIC materials prepared following the i-CONSENT guidelines. Additional objectives are to (1) analyze differences in comprehension across countries (Spain, the United Kingdom, and Romania) and evaluate whether materials designed for one region apply to other regions or languages; (2) assess comprehension within specific domains of IC information; and (3) evaluate participant satisfaction with the eIC materials. Additional objectives for adults include exploring differences in comprehension by gender and age, while for minors, the objective is to explore differences in comprehension by gender.

## Methods

### Elaboration of e-Consent Materials

#### Overview

Electronic information materials have been prepared for 3 mock studies on vaccine clinical trials, each addressed to a different population group (see Table 1).

**Table 1.** Description of the 3 case studies.

Variable	Minors	Pregnant women	Adults
Target population			
Age (years)	12-13	≥18	Millennials: 18‐38; Generation X: 39‐53
Genders included	Both male and female	N/A^a^	Both male and female
Sample size, n	620	312	825
Case scenarios	Human papillomavirus vaccination in minors, considering gender differences.	Respiratory syncytial virus vaccination in pregnant women, considering both their needs and those of their babies.	Meningococcal vaccination in adults, considering gender and generational differences.
How was the target population involved?	One design thinking session with children and parents, 1 session with children alone, and piloting of the contents of the information sheet and the survey.	Two design thinking sessions with pregnant women and piloting of materials.	An online-based self-administered survey used for piloting.
Materials prepared	eConsent^b^, including information in 3 formats: a website page (layered approach), a video (storytelling format), and a document (improved format).	eConsent included information in 3 formats: a website page (layered approach), 1 infographic (about the study procedures), a video (question-and-answer format), and a document (improved format).	eConsent included information in 3 formats: a website page (layered approach), 5 infographics (covering general information, procedures, benefits and risks, legal aspects, and data protection), and a document (improved format).

a N/A: not applicable.

b eConsent: electronic consent.

The materials have been prepared following the i-CONSENT guidelines, taking into account potential participants’ preferences and needs [4]. Different formats were included, and participants could choose among these formats or combine them. Table 1 provides information about the materials prepared.

#### Development Process

The materials were originally prepared in Spanish through a cocreation process involving representatives from the target population. A multidisciplinary team comprising clinical trial physicians, epidemiologists, a sociologist, a journalist, and a nurse collaborated on the design. This approach ensured that the materials were scientifically accurate while addressing the cognitive and cultural needs of participants. The cocreation methodology included participatory sessions with minors and pregnant women in Spain to ensure that the materials were relevant and engaging for these groups. For adults, online surveys provided insights into preferences and usability. This iterative process allowed for refinement based on user feedback before finalizing the materials.

To facilitate cross-cultural implementation, the materials were professionally translated into English and Romanian by native speakers of the target languages. The translation process adhered to a rigorous rubric that prioritized fidelity to meaning, contextual appropriateness, and adaptation to local customs and linguistic conventions. Each translation was independently reviewed by another professional translator to ensure quality and consistency.

#### Formats Offered

Participants accessed the eIC materials via a dedicated website, where they could choose from multiple formats based on their preferences or combine them as needed. The formats offered (depending on the specific case study) included the following:

- Layered web content: A modular approach that allowed participants to access additional details or definitions by clicking on specific terms.
- Narrative videos: Tailored video formats for minors and pregnant women (a question-and-answer style for pregnant women and narrative storytelling for minors).
- Printable documents: Text-based materials with integrated images, designed for participants who prefer physical copies.
- Infographics: Simplified visual representations of complex topics such as legal aspects or participant rights.

The formats were not mutually exclusive, and participants could engage with any combination of formats according to their preferences.

### Comprehension Assessment Tool

#### Adaptation of the Quality of the Informed Consent Questionnaire for Target Populations

To evaluate comprehension, 3 tailored adaptations of the Quality of the Informed Consent questionnaire (QuIC) [5] were used, 1 for each mock study. The adaptations were based on the original version of the QuiC developed by Joffe et al [5] and subsequent modifications by Paris et al [6,7]. The adjustments accounted for the nature of the study (vaccine clinical trials in healthy populations), the characteristics of the target groups (minors, pregnant women, and adults), and European regulations on data protection and clinical trials.

#### Development and Validation

Each adaptation process was conducted independently for the respective population. For minors and pregnant women, cocreation sessions were held with representatives from these groups to ensure that the questionnaires were appropriate and comprehensible. Feedback from these sessions informed iterative revisions, resulting in the final versions of the tools. The adapted questionnaires were originally developed in Spanish and subsequently translated into English and Romanian by professional translators. These translations followed a rigorous rubric that prioritized fidelity to meaning, cultural appropriateness, and linguistic clarity.

#### Structure of the Questionnaires

The surveys, as their predecessors, consist of 2 parts to assess understanding: part A measures objective understanding, and part B measures subjective understanding.

In the surveys for adults and pregnant women, part A includes 22 questions with 3 response options (“no,” “don’t know,” and “yes”). The scoring system follows that of the original version of the QuIC [5]: 0 points for an incorrect answer, 50 points for a “don’t know” response, 100 points for a correct answer. The questions in part A are grouped into 7 domains: “Nature/purposes of research,” “Risks and benefits,” “Alternative procedures,” “Legal, participant protection,” “Compensation,” “Contacts, information,” and “Participants’ rights.” Part B consists of 13 questions scored using a 5-point Likert scale. Scores are assigned as follows: 0 points for a rating of 1, 25 points for 2, 50 points for 3, 75 points for 4, and 100 points for 5.

The survey to measure children’s understanding was designed in a cocreation session with children and resulted in the Assent Comprehension Questionnaire for vaccine studies (abbreviated *CCAsIn*, from its Spanish title *Cuestionario de Comprensión del Asentimiento Informado*) [4]. In this survey, part A includes 14 questions with 2 response options (“agree” and “disagree”); correct answers score 100 points, and incorrect answers score 0 points. Part B consists of 10 questions, scored using the same values as in the adult version. The minors’ survey includes 3 domains: “Nature/purposes of research,” “Risks and benefits,” and “Participants’ rights.”

The global score of each part was calculated as the average score of all questions in that part. Domain-specific scores were also calculated for part A by averaging the scores within each domain.

Additionally, 2 sections were added: 1 with sociodemographic questions and 1 with questions addressing acceptance, preferences, and satisfaction with the eIC materials. A multiple-choice question was included to capture participants’ preferences regarding information formats.

The final versions of the questionnaires underwent pilot testing with representatives from each target population to ensure validity and reliability. Feedback from participants informed minor adjustments before implementation in the main study.

Parts A and B of each survey are included in Multimedia Appendix 1, along with the frequency of responses for each question. Multimedia Appendix 1 also presents the questions grouped by domain.

### Ethical Considerations

#### Ethics Review and Approvals

This study was conducted in accordance with the Declaration of Helsinki and received ethical approval from the Research Ethics Committee of the Foundation for the Promotion of Health and Biomedical Research of the Valencia Region (FISABIO; approval number 20200109/09).

#### Informed Consent

All participants provided IC before participation (see Multimedia Appendix 1). For minors, parental/guardian consent was obtained alongside assent from the children themselves. Online participants provided consent/assent electronically via a digital form integrated into the survey platform. Participants were explicitly informed that their involvement was voluntary and not related to any actual clinical trial.

#### Privacy and Confidentiality

All data collected were anonymized and securely stored on password-protected servers. Personal identifiers were removed before analysis, and participants were assured that their responses would remain confidential. Access to the raw data was restricted to the research team.

#### Compensation

Participants received compensation based on their recruitment group:

- Minors in Spain: Schools where the surveys were conducted received an Amazon voucher worth €200 (US $235) as an institutional incentive. For minors outside Spain, no direct compensation was provided due to logistical constraints; instead, recruitment was facilitated by the market research company (GfK) that visited schools to oversee participation.
- Adults and pregnant women: Participants recruited through the market research company’s panel database received points equivalent to €4 to €5 (US $5 to US $6), which could be redeemed for gift cards or other rewards through the panel’s system.

### Recruitment

#### Rationale for Selecting Target Populations

The study targeted 3 distinct populations: minors, pregnant women, and adults. These groups were selected due to their specific ethical and regulatory considerations in vaccine clinical trials. Minors were included because their participation requires both assent and parental authorization, presenting ethical challenges due to their classification as a vulnerable population. Pregnant women were chosen to examine ethical safeguards and risk perceptions that may influence their willingness to participate, particularly in the context of vaccines administered during pregnancy to protect the unborn child. Adults were included as they represent the primary target population for vaccine trials, allowing for an assessment of IC comprehension and decision-making within a generally healthy population, as well as enabling intergenerational comparisons.

Surveys were conducted in 3 countries —Spain, the United Kingdom, and Romania—through the market research company with established household panel databases (30,000 panelists in Spain, 700,000 in Romania, and 2 million in the United Kingdom). The selection of these countries followed a cluster sampling approach based on their geographical distribution across Northern, Southern, and Eastern Europe. Factors such as cultural and linguistic diversity, country size, the number of registered clinical trials, and affiliation with the consortium were considered in the selection process.

#### Recruitment of Minors

For minors, recruitment was initially intended to be conducted face-to-face in schools. However, due to COVID-19 restrictions, this approach was adapted to online surveys in the United Kingdom and Romania. In Spain, surveys were partially conducted at state-subsidized schools under controlled conditions and complemented by online surveys completed at home. To ensure comparability across countries, recruitment targeted children aged 12‐13 attending state-subsidized schools or equivalent educational settings.

A recruitment questionnaire was sent to randomly selected panelists with children aged 12 or 13 years. Parents were asked whether their children attended subsidized schools; if they responded affirmatively, they were invited to participate. Once parental IC was obtained, children were asked to provide their assent to participate. Parents also signed a certification confirming that survey responses were provided by their children rather than themselves. Materials and surveys were then sent online to participants who agreed to participate.

In Spain, 187 out of 312 students completed the survey in information technology classrooms under the supervision of study representatives, while the remainder participated from home. The data obtained across all 3 countries were fully comparable, as the sample profiles of children were matched across regions and the surveys that were conducted using similar procedures.

#### Recruitment of Adults and Pregnant Women

Adults and pregnant women were recruited using the market research company’s household panel database for online studies. Invitations were sent to randomized samples of panelists who met the age and sex criteria. Sampling was controlled by area, age group, and socioeconomic level to ensure representativeness.

To identify pregnant women, the recruitment questionnaire included the question, “Are you currently pregnant?” All women who replied in the affirmative were included in the study group for pregnant women.

In Spain, due to the smaller panel size for pregnant women and the larger sample requirements, part of the recruitment was conducted face-to-face near health centers in Valencia and Madrid. This was carried out by professional recruiters using convenience sampling criteria. Pregnant women approached by recruiters were asked whether they wished to participate after being presented with the study details. Interested participants provided their email addresses via a dedicated form for follow-up communication. Subsequently, documentation and participation links were sent electronically in the same manner as for other panelists.

#### Instructions Provided to Participants

Participants received materials via email along with a standardized script explaining the study’s purpose and procedures. This script included (1) an introduction to FISABIO’s research within the framework of the European i-CONSENT project; (2) a statement clarifying that participation was voluntary and unrelated to any actual clinical trial; (3) an overview of the study structure (reviewing IC materials followed by a survey); and (4) estimated times for viewing the materials (35-40 minutes) and completing the survey (a minimum of 12 minutes).

Participants were instructed to review all materials before completing the survey. They accessed the materials via a private link sent to their email or provided during recruitment. The website allowed participants to open the materials in a browser tab while completing the survey in another, enabling them to refer back to the information as needed during data collection.

#### Fieldwork Period

All fieldwork for all study groups and countries was conducted between September and October 2020.

#### Survey Platform and Data Management

The surveys were administered using Confirmit Horizons v24, a multimodal platform employed by GfK for data collection via computer-assisted telephone interviewing, computer-assisted personal interviewing, or computer-assisted web interviewing methodologies. Data were securely transmitted from the platform to the research team, ensuring anonymization and compliance with data protection standards.

#### Adaptations Due to the COVID-19 Pandemic

A pilot test was conducted in Spain in July 2020 to evaluate the feasibility of face-to-face recruitment. The results (530 people approached, 467 uninterested, 5 completed surveys on-site, and 58 completed surveys from home via email) highlighted significant challenges with in-person recruitment and demonstrated the practicality of online data collection methods. Consequently, all surveys were conducted online, except for minors in Spain, where a hybrid approach was used (see the CHERRIES [Checklist for Reporting Results of Internet E-Surveys] checklist in Multimedia Appendix 1).

### Statistical Analysis

#### Summary of Descriptive Statistics

Descriptive statistics were calculated for the study database, stratified by country, age, and gender (except for pregnant women, where gender was not applicable). Categorical variables were summarized using frequencies and percentages, while quantitative variables were described using means and SDs.

#### Comprehension Score

Comprehension of the study materials was assessed using the total score from part A of the adapted QuIC surveys, as well as by individual domains of IC. Scores were categorized as follows, based on the maximum score: below 70% indicated low comprehension; between 70% and 79% indicated moderate comprehension; between 80% and 89% indicated adequate comprehension; and 90% or higher indicated high comprehension.

These cut-off points were established based on previous studies [8-10].

#### Regression Modeling

To evaluate differences in comprehension scores (part A) by country, age (adults), or gender (adults and minors), a linear regression model was constructed. Given the exploratory nature of the objectives, the model was adjusted for age (adults only), country, gender, previous participation in a clinical trial, and educational level (up to primary vs tertiary education, adults only), as these factors were anticipated to be strong predictors of comprehension. Interactions between variables were tested, and the Akaike information criterion (AIC) was used to select the optimal model by penalizing for model complexity. As the mean score did not follow a normal distribution, an ordered quantile normalizing transformation was applied to the data before regression analysis [11].

The analysis was carried out using the statistical software R, version 4.0.3 (R Foundation).

#### Data Exclusion Criteria

Participants whose response pattern indicated that more than 80% of items in the part A questionnaire were answered as “don’t know” were excluded from the analyses (n=6).

## Results

### Study Sample and Participant Characteristics

After exclusions, a total of 1757 participants were included in the analysis: 825 adults, 312 pregnant women, and 620 minors. Key sociodemographic characteristics are summarized in Tables 2-4.

**Table 2.** Characteristics of the minors (n=620).

Characteristics	Spain (n=312), n (%)	Romania (n=125), n (%)	United Kingdom (n=183), n (%)	All (n=620), n (%)
Age				
12 years old	149 (47.8)	72 (57.6)	103 (56.3)	324 (52.3)
13 years old	163 (52.2)	53 (42.4)	80 (43.7)	296 (47.7)
Gender				
Male	167 (53.5)	47 (37.6)	88 (48.1)	302 (48.7)
Female	145 (46.5)	78 (62.4)	95 (51.9)	318 (51.3)
Previous participation in a real clinical trial				
No	293 (93.9)	118 (94.4)	176 (96.2)	587 (94.7)
Yes	19 (6.1)	7 (5.60)	7 (3.8)	33 (5.3)

**Table 3.** Characteristics of the pregnant women (n=312).

Characteristics	Spain (n=163), n (%)	Romania (n=89), n (%)	United Kingdom (n=60), n (%)	All (n=312), n (%)
Age groups				
<29 years old	41 (25.2)	30 (33.7)	28 (46.7)	99 (31.7)
29‐38 years old	100 (61.3)	47 (52.8)	29 (48.3)	176 (56.4)
39‐45 years old	18 (11.0)	12 (13.5)	3 (5.0)	33 (10.6)
>45 years old	4 (2.5)	0 (0)	0 (0)	4 (1.3)
Previous participation in a real clinical trial				
No	142 (87.1)	76 (85.4)	45 (75.0)	263 (84.3)
Yes	21 (12.9)	13 (14.6)	15 (25.0)	49 (15.7)
Educational level				
None completed/primary school/high school	52 (31.9)	17 (19.1)	10 (16.7)	79 (25.3)
University or higher	111 (68.1)	72 (80.9)	50 (83.3)	233 (74.7)

**Table 4.** Characteristics of the adults (n=825)

Characteristics	Spain (n=420), n (%)	Romania (n=245), n (%)	United Kingdom (n=160), n (%)	All (n=825), n (%)
Gender				
Male	219 (52.1)	122 (49.8)	79 (49.4)	420 (50.9)
Female	201 (47.9)	123 (50.2)	81 (50.6)	405 (49.1)
Age group				
<29 years old	105 (25.0)	57 (23.3)	37 (23.1)	199 (24.1)
29‐38 years old	112 (26.7)	63 (25.7)	43 (26.9)	218 (26.4)
39‐45 years old	99 (23.6)	65 (26.5)	40 (25.0)	204 (24.7)
>45 years old	104 (24.8)	60 (24.5)	40 (25.0)	204 (24.7)
Previous participation in a real clinical trial				
No	401 (95.5)	230 (93.9)	134 (83.8)	765 (92.7)
Yes	19 (4.5)	15 (6.1)	26 (16.3)	60 (7.3)
Educational level				
None completed/primary school/high school	179 (42.6)	23 (9.4)	46 (28.8)	248 (30.1)
University or higher	241 (57.4)	222 (90.6)	114 (71.3)	577 (69.9)

### Objective Comprehension (Part A)

#### Comprehension Outcomes by Population Group

The mean objective comprehension scores (adapted QuIC part A) were high across all groups: minors, mean 83.3 (SD 13.5); pregnant women, mean 82.2 (SD 11.0); and adults, mean 84.8 (SD 10.8). All domains achieved mean scores above 70%, with no domains falling below 80% in any of the target populations (Table 5). The distribution of comprehension levels is presented in Table 6.

**Table 5.** Mean scores and SDs of each domain by study case (adapted QuIC^a^ part A).

Domain	Minors, mean (SD)	Pregnant women, mean (SD)	Adults, mean (SD)
Nature/purpose of research	84.3 (16.2)	85.8 (17.3)	85.7 (15.3)
Risks and benefits	85.4 (13.2)	70.5 (21.4)	82.8 (19.3)
Alternative procedures	N/A^b^	90.2 (24.9)	77.0 (23.4)
Legal, participant protection	N/A	78.9 (19.9)	87.3 (15.6)
Compensation	N/A	84.0 (20.3)	82.7 (21.3)
Contacts, information	N/A	80.8 (16.4)	79.2 (20.7)
Participants’ rights	88.8 (23.2)^c^	90.0 (14.7)	92.3 (13.0)
Final score for QuIC part A	83.3 (13.5)	82.2 (11.0)	84.8 (10.8)

a QuIC: Quality of the Informed Consent questionnaire.

b N/A: not applicable.

c In the minors’ survey “Contacts, information” and “Participants’ rights” were grouped into a single domain, as there was only 1 question on “Contacts, information” (A12), which made it very weak for analysis.

**Table 6.** Distribution of participants in each level of comprehension by case study (N=1757).

Degree of comprehension (part A)	Minors (n=620), n (%)	Pregnant (n=312), n (%)	Adults (n=825), n (%)
High (≥90%)	259 (41.8)	91 (29.2)	322 (39.0)
Adequate (≥80% and <90%)	109 (17.6)	100 (32.1)	287 (34.8)
Moderate (≥70% and <80%)	153 (24.7)	80 (25.6)	117 (14.2)
Low (<70%)	99 (16.0)	41 (13.1)	99 (12.0)

#### Factors That Have an Influence on the Understanding

Regression analyses identified several predictors of comprehension: (1) in minors, lower scores were associated with being male, aged 13, non-Spanish, and having prior clinical trial participation (Table 7); (2) among pregnant women, lower scores were linked to previous trial participation and lower education levels, particularly in Romania (Table 8); and (3) for adults, higher scores were observed among women, members of Generation X, Spanish participants, those with higher education, and those without prior trial participation (Table 9).

**Table 7.** Association between adapted QuIC^a^ part A and covariates: minors.

Variables	Univariable model, β coefficient (95% CI); *P* value	Multivariable model, β coefficient (95% CI); *P* value
Gender		
Boy	N/A^b^	N/A
Girl	0.32 (0.18 to 0.47); <.001	0.36 (0.22 to 0.50); <.001
Age		
12 years old	N/A	N/A
13 years old	−0.17 (−0.32 to −0.02); .02	−0.20 (−0.34 to −0.06); .006
Which country do you live in?/Previous participation in a clinical trial		
Spain/No	N/A	N/A
Spain/Yes	−0.52 (−0.95 to −0.10); .01	−0.46 (−0.87 to −0.04); .03
Romania/No	−0.18 (−0.38 to 0.01); .06	−0.26 (−0.45 to −0.07); .009
Romania/Yes	−1.71 (−2.40 to −1.03); <.001	−1.77 (−2.43 to −1.10); <.001
United Kingdom/No	−0.25 (−0.42 to −0.08); .004	−0.29 (−0.45 to −0.12); .001
United Kingdom/Yes	−1.37 (−2.05 to −0.69); <.001	−1.34 (−2.01 to −0.67); <.001

a QuIC: Quality of the Informed Consent questionnaire.

b N/A: not applicable.

**Table 8.** Association between adapted QuIC^a^ part A and covariates: pregnant women.

Variables	Univariable model, β coefficient (95% CI); *P* value	Multivariable model, β coefficient (95% CI); *P* value
Generation		
Millennials	N/A^b^	N/A
Generation X	0.25 (−0.09 to 0.59); .15	0.21 (−0.12 to 0.54); .22
Previous participation in a clinical trial		
No	N/A	N/A
Yes	−0.71 (−1.00 to −0.41); <.001	−0.68 (−0.97 to −0.39); <.001
Which country do you live in? (Educational level)		
Spain (none completed/primary school/high school)	N/A	N/A
Spain (university or higher education)	0.06 (−0.26 to 0.38); .72	0.03 (−0.28 to 0.34); .87
United Kingdom (none completed/primary school/high school)	−0.16 (−0.69 to 0.38); .56	−0.26 (−0.78 to 0.26); .33
United Kingdom (university or higher education)	−0.03 (−0.38 to 0.32); .86	−0.02 (−0.36 to 0.32); .91
Romania (none completed/primary school/high school)	−1.15 (−1.81 to −0.49); .001	−1.05 (−1.69 to −0.41); .001
Romania (university or higher education)	0.12 (−0.26 to 0.50); .53	0.20 (−0.17 to 0.56); .30

a QuIC: Quality of the Informed Consent questionnaire.

b N/A: not applicable.

**Table 9.** Association between adapted QuIC^a^ part A and covariates: adults.

Factors	Univariable model, β coefficient (95% CI); *P* value	Multivariable model, β coefficient (95% CI); *P* value
Gender		
Male	N/A^b^	N/A
Female	0.18 (0.04 to 0.31); .01	0.16 (0.03 to 0.29); .02
Generation		
Millennials	N/A	N/A
Generation X	0.25 (0.12 to 0.38); <.001	0.26 (0.12 to 0.39); <.001
Which country do you live in?		
Spain	N/A	N/A
United Kingdom	−0.19 (−0.34 to −0.04); .02	−0.27 (−0.43 to −0.11); .001
Romania	−0.27 (−0.45 to −0.09); .003	−0.25 (−0.43 to −0.07); .005
Educational level		
None completed/primary school/high school	N/A	N/A
University or higher education	0.15 (0 to 0.30); .04	0.22 (0.07 to 0.38); .004
Previous participation in a clinical trial		
No	N/A	N/A
Yes	−0.58 (−0.84 to −0.33); <.001	−0.47 (−0.73 to −0.21); <.001

a QuIC: Quality of the Informed Consent questionnaire.

b N/A: not applicable.

Materials cocreated in Spain were effective across all countries, although comprehension was generally higher in the original target population. Lower educational levels in Romania were associated with reduced comprehension.

### Subjective Comprehension (Part B)

#### Comprehension Outcomes by Population Group

This was measured using a 5-point Likert scale ranging from 1 (“I did not understand anything”) to 5 (“I understood everything”), and a final score was calculated. Subjective comprehension was high across all groups, with mean scores exceeding 85 out of 100 (Table 10).

**Table 10.** Mean scores and SDs by study case (adapted QuIC^a^ part B).

Study case	Minors, mean (SD)	Pregnant women, mean (SD)	Adults, mean (SD)
B1	4.31 (0.81)	4.56 (0.72)	4.60 (0.72)
B2	4.68 (0.66)	4.73 (0.62)	4.81 (0.53)
B3	4.38 (0.87)	4.50 (0.82)	4.50 (0.83)
B4	4.42 (0.79)	4.57 (0.70)	4.50 (0.79)
B5	4.49 (0.79)	4.44 (0.90)	4.42 (0.85)
B6	4.16 (0.98)	4.35 (0.81)	4.54 (0.78)
B7	4.39 (0.78)	4.33 (0.86)	4.43 (0.83)
B8	4.07 (1.07)	4.29 (0.91)	4.47 (0.84)
B9	4.54 (0.72)	4.58 (0.67)	4.61 (0.70)
B10	4.66 (0.66)	4.51 (0.72)	4.46 (0.86)
B11	N/A^b^	4.51 (0.81)	4.57 (0.75)
B12	N/A	4.77 (0.54)	4.76 (0.58)
B13	N/A	4.56 (0.63)	4.56 (0.65)
Total score (QuIC part B)	85.2 (14.1)	87.9 (13.7)	88.9 (13.2)

a QuIC: Quality of the Informed Consent questionnaire.

b N/A: not applicable.

#### Preferences and Satisfaction

Minors and pregnant women preferred video, while adults favored text (Table 11). Satisfaction was high, with over 90% of participants in all groups (minors, 604/620, 97.4%; pregnant women, 303/312, 97.1%; and adults, 804/825, 97.5%) reporting that the materials were easy to understand and helpful (Table 12).

**Table 11.** Preferred information formats by target population.

How would you prefer to be given the information if you were to participate in a clinical trial? (You may choose various answers)	Minors (n=620), n (%)	Pregnant women (n=312), n (%)	Adults (n=825), n (%)
Written text on paper			
No	438 (70.6)	166 (53.2)	373 (45.2)
Yes	182 (29.4)	146 (46.8)	452 (54.8)
Web			
No	385 (62.1)	210 (67.3)	397 (48.1)
Yes	235 (37.9)	102 (32.7)	428 (51.9)
Video			
No	238 (38.4)	160 (51.3)	586 (71.0)
Yes	382 (61.6)	152 (48.7)	239 (29.0)
To be told by someone in charge of the trial			
No	488 (78.7)	213 (68.3)	457 (55.4)
Yes	132 (21.3)	99 (31.7)	368 (44.6)

**Table 12.** Satisfaction and perceived difficulty of information by the target population.

Question	Minors (n=620), n (%)	Pregnant (n=312), n (%)	Adults (n=825), n (%)
What is your overall satisfaction with the information you have read/seen?			
Not at all satisfied/not happy at all^a^	16 (2.6)	9 (2.9)	21 (2.5)
Satisfied/happy^a^	358 (57.7)	188 (60.3)	466 (56.5)
Very satisfied/very happy^a^	246 (39.7)	115 (36.9)	338 (41.0)
Has the information you have read/seen helped you to understand the clinical trial?			
No	16 (2.6)	5 (1.6)	8 (1.0)
Somewhat/a bit^a^	98 (15.8)	17 (5.4)	40 (4.8)
Yes	506 (81.6)	290 (92.9)	777 (94.2)
What is your impression of the information you have read/seen in terms of understanding?			
Very difficult	9 (1.5)	6 (1.9)	7 (0.8)
Difficult	44 (7.1)	23 (7.4)	64 (7.8)
Easy	410 (66.1)	201 (64.4)	526 (63.8)
Very easy	157 (25.3)	82 (26.3)	228 (27.6)
Have you felt the need to ask questions about the clinical trial to the medical professional who appeared in the video/informational materials?			
No	N/A^b^	189 (60.6)	604 (73.2)
Yes	N/A	123 (39.4)	221 (26.8)

a Wording used in the minor’s survey.

b N/A: not applicable.

## Discussion

### Principal Findings

This exploratory, cross-sectional study provides preliminary evidence that eIC materials developed according to the i-CONSENT guidelines may be associated with high levels of comprehension and satisfaction among minors, pregnant women, and adults in 3 European countries. Objective comprehension scores (part A) exceeded 80% in all groups, and subjective comprehension (part B) was similarly high. However, a discrepancy was observed between subjective and objective understanding, consistent with previous research using QuIC-based tools [6,12-16]. This suggests that self-perceived understanding may not always reflect actual comprehension, underscoring the importance of ongoing assessment and targeted improvements in consent materials.

Minors exhibited greater variability in comprehension scores, with a higher proportion of both high (≥90%) and low (<70%) results compared with other groups. This finding highlights the challenges in tailoring materials for younger populations and suggests that additional support may be needed to facilitate their understanding. Certain domains, such as randomization and placebo concepts, remained difficult to understand for all groups, consistent with prior studies [1,14,17].

Notably, some survey questions yielded poor comprehension rates, particularly those requiring a “disagree” response or containing negative phrasing, consistent with findings from the original QuIC questionnaire [5]. Negatively worded items are known to increase cognitive load and reduce response accuracy [18]. Although these questions aimed to assess understanding of critical concepts such as randomization and placebo, their structure may have contributed to the observed difficulties. In the case of minors, the questionnaire was cocreated with children to ensure suitability [4], making it difficult to determine whether the phrasing of the questions or the complexity of the concepts was the primary factor influencing comprehension.

Formats designed using a layered approach allowed participants to engage with content according to their needs and preferences, which may have contributed to the overall high satisfaction and comprehension rates. Format preferences varied by population: minors and pregnant women tended to prefer video, while adults favored written text. These findings are consistent with previous research suggesting that offering multiple formats can accommodate diverse learning styles and improve engagement [19,20]. The separation of information delivery from discussion with the investigator was reinforced, aligning with literature indicating that combining both methods—especially when accompanied by extended discussion—can further enhance understanding [21].

Satisfaction with the materials was high across all groups, with 1604 out of 1757 (91.29%) participants rating the materials as easy or very easy to understand, and fewer than 3% (46/1757, 2.62%) expressing dissatisfaction. These results suggest that the cocreation process, which actively involved representatives from each target population, contributed to the development of user-friendly materials tailored to diverse needs [3,4,19]. The integration of multimedia tools, including layered content presentation and interactive features, likely supported engagement and comprehension [19], although the impact of these features on actual decision-making remains to be further explored. It is also important to consider the potential limitations of digital resources, particularly for populations with limited access to technology or low digital literacy, as these factors could influence both comprehension and satisfaction outcomes [22,23].

### Comparison With Previous Work

The results of this exploratory study are consistent with previous research indicating that structured, multimodal information materials can enhance participant comprehension and satisfaction in the IC process. Compared with the original QuIC study by Joffe et al [5], which reported a mean objective score of 77.8, our study achieved higher mean scores across all target populations (ranging from 82 to 84.8), while subjective comprehension scores remained similar (mean 85.2‐88.9). This suggests that the application of the i-CONSENT guidelines, particularly the use of layered and cocreated materials, may contribute to improved understanding without inflating self-perceived comprehension.

The layered approach and the inclusion of multiple formats (text, video, infographics) align with recommendations from the i-CONSENT guidelines and recent systematic reviews, which highlight the importance of tailoring information to participant needs and preferences to address health literacy barriers and support informed decision-making [3,4,19]. Our findings reinforce the value of cocreation, as actively involving representatives from the target population in the development of materials has been shown to increase relevance, accessibility, and user satisfaction [4]. This is consistent with the i-CONSENT project’s emphasis on participatory design and the RAND/UCLA (University of California, Los Angeles) expert consensus, which identified cocreation and a layered approach as key strategies for improving IC [3].

Demographic factors such as gender, age, and education were significant predictors of comprehension, consistent with previous literature. Women consistently achieved higher comprehension scores than men, a trend also observed by Raich et al [24] and Tam et al [1]. Age-related differences were apparent: older adults generally outperformed younger adults, in line with findings by Klima et al [25] and Tam et al [1]. By contrast, among minors, older participants scored lower than their younger peers, suggesting that factors such as motivation or engagement may influence comprehension in this subgroup and warrant further investigation [26].

A notable and unexpected finding was the negative association between prior clinical trial participation and comprehension. While some previous studies have suggested that experience in clinical trials enhances understanding [22,25], our results indicate that returning participants may overlook critical information due to perceived familiarity, potentially leading to overconfidence and knowledge gaps. Given the relatively small proportion of prior participants in our sample (adults, 60/825, 7.3%; pregnant women, 49/312, 15.7%; and minors, 33/620, 5.3%), this trend should be interpreted with caution. Future research should explore whether repeated exposure to trial materials influences learning patterns or whether targeted interventions can address overconfidence in returning participants.

Socioeconomic and cultural factors also played a role in comprehension outcomes. Higher education attainment was associated with better understanding, as reported in previous studies [1,16,22,27], yet participants with lower education still achieved reasonable scores, suggesting that the design of the materials may have mitigated some barriers related to health literacy. Comprehension remained high (>80%) in translated materials, consistent with Addissie et al [22], indicating that cocreated materials developed in one country can be effectively utilized in others. However, the observed differences in comprehension between Spain and other countries suggest that further adaptation to local linguistic and cultural contexts may be beneficial to optimize understanding across all populations.

Format preferences also influenced comprehension. The preference for video formats among minors and pregnant women aligns with Gesualdo et al [19], while adults’ preference for text-based materials is consistent with health literacy principles described by Lorenzen et al [28]. These findings underscore the importance of offering multiple formats to accommodate diverse learning styles and ensure accessibility for all participants.

### Strengths and Limitations

#### Overview

This section presents the main strengths that support the validity and applicability of the findings, as well as the limitations that should be taken into account to interpret the results and guide future research.

#### Strengths

One of the key strengths of this study is its multinational design, which evaluated eIC materials across 3 distinct cultural contexts—Spain, the United Kingdom, and Romania. This approach enhances the external validity of the findings and demonstrates the potential applicability of the i-CONSENT guidelines in diverse settings. The large sample size (1757 participants) and the inclusion of 3 target populations—minors, pregnant women, and adults—allowed for robust subgroup analyses and increased the generalizability of the results.

The cocreation process, which actively involved representatives from each target population, is another notable strength. This participatory methodology ensured that the materials were tailored to real-world needs and preferences, likely contributing to the high satisfaction and comprehension rates observed [3]. The use of multiple content formats (layered web content, videos, printable documents, and infographics) provided participants with flexibility in how they accessed information, accommodating different learning styles and potentially reducing barriers related to health literacy [19].

The study also benefited from the use of adapted versions of the QuIC questionnaire, which enabled a comprehensive assessment of both objective and subjective understanding. This dual assessment helped identify specific comprehension challenges, allowing for targeted recommendations for improvement [5]. For minors, the survey was codeveloped with children, further enhancing its relevance and appropriateness for this group [4].

#### Limitations

Several limitations should be considered when interpreting these findings. First, the study used a cross-sectional design without a control group, which limits the ability to attribute observed outcomes directly to the i-CONSENT guidelines.

Second, the materials tested represent only 3 specific examples of eIC products developed according to the i-CONSENT guidelines. While these materials were tailored to the needs of minors, pregnant women, and adults, they do not encompass the full range of possible designs or clinical contexts. Additionally, participant engagement with specific formats was not monitored, so the impact of each format on comprehension could not be directly assessed.

Third, recruitment was conducted primarily online due to COVID-19 restrictions, which may have excluded individuals with limited digital access or lower digital literacy. Although printable documents were offered as an alternative format to mitigate this limitation, the sample may not fully represent populations with the greatest barriers to digital participation.

Fourth, while professional translators prepared the English and Romanian versions of the materials, end-user validation of translations was not performed. Although the translations were reviewed by native speakers within the consortium, the lack of direct feedback from target users may have affected comprehension, particularly in non-Spanish populations. Prior studies have highlighted that lower education levels can negatively impact comprehension of IC materials [22], and this effect has been particularly noted among Romanian participants [23].

Finally, the proportion of participants with prior clinical trial experience was relatively small, which may limit the interpretation of findings related to this subgroup. The exploratory nature of the study and the specific sample characteristics further limit the generalizability of the results.

### Future Directions

Building on the exploratory findings of this study, several avenues for future research are warranted to further advance the development and implementation of IC materials tailored to diverse populations.

First, additional research should investigate the underlying causes of the polarized comprehension scores among minors and the negative association between prior clinical trial participation and understanding. Qualitative studies or mixed-method approaches could help elucidate whether factors such as motivation, engagement, or overconfidence contribute to these trends. Interventions designed to address these factors, such as interactive modules, adaptive content, or targeted reminders, should be evaluated for their effectiveness in improving comprehension, particularly among returning participants.

Second, the impact of multimedia formats on comprehension and satisfaction merits further exploration. While video content was preferred by minors and pregnant women, its overall effectiveness in improving comprehension remains unclear [19]. Future studies could apply experimental designs, user engagement analytics, or eye-tracking methodologies to assess how participants interact with various formats and which features most effectively support comprehension and retention. Additionally, the role of specific multimedia elements, such as animations or interactive elements, should be systematically evaluated.

Third, the generalizability of cocreated materials across broader cultural and linguistic contexts requires further validation. Although this study demonstrated that materials cocreated in one country can be applied in others, local adaptation and end-user validation may be necessary to optimize comprehension, especially in populations with lower educational attainment or limited health literacy. Comparative studies across additional countries and languages, as well as research on the impact of different translation and adaptation strategies, would provide valuable insights.

Finally, longitudinal research is needed to assess whether improved comprehension at the time of consent translates into better retention of information and more informed decision-making over the course of clinical trial participation. Such studies could also explore the long-term effects of digital consent tools on participant engagement, satisfaction, and trust in research.

### Conclusions

This exploratory study provides preliminary evidence supporting the effectiveness of eIC materials developed according to the i-CONSENT guidelines in enhancing comprehension and satisfaction among diverse populations. Across 3 countries—Spain, the United Kingdom, and Romania—and 3 target groups—minors, pregnant women, and adults—objective comprehension scores consistently exceeded 80%, while subjective comprehension and satisfaction rates surpassed 90%. These findings highlight the potential of cocreated, multimodal consent materials to address diverse participant needs and preferences.

Demographic factors such as gender, age, and educational attainment significantly influenced comprehension outcomes. Women consistently outperformed men, and older adults achieved higher scores than younger ones. However, the negative association between prior clinical trial participation and comprehension underscores the need for targeted strategies to engage returning participants and address potential overconfidence or reduced attention to consent materials. These findings call for further investigation into how prior experience shapes participant behavior during the IC process.

The study also demonstrated that materials cocreated in one cultural context can be effectively applied in others, provided that cultural and linguistic adaptations are carefully implemented. However, differences in comprehension scores between countries suggest that additional localization efforts may further optimize outcomes. Format preferences varied across populations, reinforcing the importance of offering multiple formats to accommodate different learning styles and align with cognitive needs.

While these results are promising, they should be interpreted with caution due to the study’s cross-sectional design and exploratory nature. The absence of a control group limits causal inference, and the findings may not be generalizable beyond the specific populations and settings studied. Future research should employ longitudinal designs to assess comprehension retention over time and explore whether improved understanding translates into more informed decision-making throughout clinical trial participation.

These results highlight the potential of user-centered design principles, such as cocreation, multimodal delivery, and cultural adaptation, to improve IC processes in clinical research. The present findings provide a foundation for further development and validation of digital consent tools across broader populations and contexts. Continued efforts to refine these approaches will be essential to ensuring that IC processes remain inclusive, effective, and responsive to participant needs.

## Supplementary material

10.2196/65569 Multimedia Appendix 1 Study questionnaires and additional documentation.

10.2196/65569 Checklist 1 CHERRIES (Checklist for Reporting Results of Internet E-Surveys).

## Abbreviations

CCAsIn: Cuestionario de Comprensión del Asentimiento Informado
CHERRIES: Checklist for Reporting Results of Internet E-Surveys
eIC: electronic informed consent
FISABIO: Foundation for the Promotion of Health and Biomedical Research of the Valencia Region
IC: informed consent
PIAAC: Program for the International Assessment of Adult Competencies
PISA: Programme for International Student Assessment
QuIC: Quality of the Informed Consent questionnaire
UCLA: University of California, Los Angeles
