# Supplementary material for: Digital Informed Consent/Assent in Clinical Trials Among Pregnant Women, Minors, and Adults: Multicountry Cross-Sectional Evaluation of Comprehension and Satisfaction
Source: JMIR Hum Factors. 2025 Aug 15;12:e65569. doi: 10.2196/65569 (PMC12356628; doi:10.2196/65569)
Supplement: Multimedia Appendix 1 [file humanfactors-v12-e65569-s001.docx]

# Multimedia Appendix 1

### I: Informed Consent Comprehension and Quality Questionnaire (c-CaCI) (adapted from the QuIC) for use in vaccine clinical trials (adult population). Parts A and B of each survey and frequency of response (correct answers are identified by a *).

**Part A**

|  | Response | | |
| --- | --- | --- | --- |
|  | DON'T KNOW | NO | YES |
| 1. One of the aims of this clinical trial is to see whether the vaccine is safe. | 34 (4.1%) | 43 (5.2%) | 748 (91%)* |
| 1. Another aim of this clinical trial is to compare the ability of the vaccine we are studying to increase defences, with respect to an already known vaccine. | 94 (11%) | 50 (6.1%) | 681 (83%)* |
| 1. An ethics committee with specialists in this field has reviewed this clinical trial and approved it. | 115 (14%) | 32 (3.9%) | 678 (82%)* |
| 1. The vaccine in the clinical trial is a way of protecting me against a disease. | 81 (9.8%) | 90 (11%) | 654 (79%)* |
| 1. When I say that I want to participate in this clinical trial, I will be placed in a group at random (for example, using heads or tails). | 86 (10%) | 59 (7.2%) | 680 (82%)* |
| 1. It has been explained to me how long the clinical trial will last. | 43 (5.2%) | 25 (3.0%) | 757 (92%)* |
| 1. My participation in this clinical trial does not entail any additional risk or discomfort. | 147 (18%) | 466 (56%)* | 212 (26%) |
| 1. If my defences are not increased by the vaccine I am given, I will have no other options. | 229 (28%) | 462 (56%)* | 134 (16%) |
| 1. Throughout the clinical trial, my doctor and I will know which group I am in. | 86 (10%) | 498 (60%)* | 241 (29%) |
| 1. It is very common to feel pain, warmth and redness at the point of injection after receiving the vaccine. | 73 (8.8%) | 81 (9.8%) | 671 (81%)* |
| 1. I have to ask all the questions I have during the visits. Afterwards it is not possible. | 84 (10%) | 642 (78%)* | 99 (12%) |
| 1. Medical and personal information is confidential and will be kept safe. | 45 (5.5%) | 9 (1.1%) | 771 (93%)* |
| 1. The sponsor has taken out a specific insurance policy for this clinical trial. | 258 (31%) | 34 (4.1%) | 533 (65%)* |
| 1. The expenses arising from my participation in the clinical trial are to be covered by me. | 74 (9.0%) | 666 (81%)* | 85 (10%) |
| 1. I am clear about who I should talk to if I have any questions or concerns about the clinical trial. | 70 (8.5%) | 22 (2.7%) | 733 (89%)* |
| 1. By participating in this clinical trial I am helping researchers gather information that can benefit others. | 30 (3.6%) | 7 (0.8%) | 788 (96%)* |
| 1. I can ask for my blood samples to be destroyed at any time. | 251 (30%) | 69 (8.4%) | 505 (61%)* |
| 1. Participation in this clinical trial is voluntary. | 24 (2.9%) | 9 (1.1%) | 792 (96%)* |
| 1. I will have to stay in the clinical trial, even if I want to leave it. | 96 (12%) | 651 (79%)* | 78 (9.5%) |
| 1. I will be given a copy of the Informed Consent document. | 118 (14%) | 10 (1.2%) | 697 (84%)* |
| 1. I cannot know the results of the clinical trial. | 338 (41%) | 339 (41%)* | 148 (18%) |
| 1. When I sign the Informed Consent for the clinical trial, I know that I agree to take part in it. | 51 (6.2%) | 12 (1.5%) | 762 (92%)* |

**Part B**

|  | Response | | | | |
| --- | --- | --- | --- | --- | --- |
|  | 1 | 2 | 3 | 4 | 5 |
| 1. That the clinical trial vaccine is being researched | 7 (0.8%) | 10 (1.2%) | 41 (5.0%) | 191 (23%) | 576 (70%) |
| 1. That my participation will help future patients. | 1 (0.1%) | 7 (0.8%) | 27 (3.3%) | 78 (9.5%) | 712 (86%) |
| 1. How long I will be in this clinical trial. | 8 (1.0%) | 16 (1.9%) | 88 (11%) | 159 (19%) | 554 (67%) |
| 1. What the researchers want to discover in the clinical trial. | 7 (0.8%) | 14 (1.7%) | 70 (8.5%) | 202 (24%) | 532 (64%) |
| 1. What I will do at each visit. | 8 (1.0%) | 21 (2.5%) | 88 (11%) | 211 (26%) | 497 (60%) |
| 1. The possible risks and inconveniences of participating in this clinical trial. | 9 (1.1%) | 13 (1.6%) | 53 (6.4%) | 196 (24%) | 554 (67%) |
| 1. The possible benefits I can obtain from participating in this clinical trial. | 8 (1.0%) | 20 (2.4%) | 76 (9.2%) | 223 (27%) | 498 (60%) |
| 1. How my personal data will be protected. | 9 (1.1%) | 18 (2.2%) | 78 (9.5%) | 188 (23%) | 532 (64%) |
| 1. How my participation may benefit the future development of vaccines that will help other people. | 5 (0.6%) | 10 (1.2%) | 42 (5.1%) | 185 (22%) | 583 (71%) |
| 1. What I should do if I become ill during the clinical trial. | 10 (1.2%) | 19 (2.3%) | 84 (10%) | 183 (22%) | 529 (64%) |
| 1. Who I should talk to if I have any questions or concerns about the clinical trial. | 4 (0.5%) | 15 (1.8%) | 59 (7.2%) | 178 (22%) | 569 (69%) |
| 1. That participation in the clinical trial is not mandatory. | 1 (0.1%) | 5 (0.6%) | 41 (5.0%) | 98 (12%) | 680 (82%) |
| 1. In general, did you understand the information about the clinical trial well? | 1 (0.1%) | 8 (1.0%) | 43 (5.2%) | 247 (30%) | 526 (64%) |

##

### II: Informed Consent Comprehension and Quality Questionnaire (c-CaCI) (adapted from the QuIC) for use in vaccine clinical trials (pregnant women). Parts A and B of each survey and frequency of response (correct answers are identified by a *).

**Part A**

|  | Response | | |
| --- | --- | --- | --- |
|  | DON'T KNOW | NO | YES |
| 1. One of the aims of this clinical trial is to see whether the vaccine is safe and effective. | 21 (6.7%) | 8 (2.6%) | 283 (91%)* |
| 1. Another aim of this clinical trial is to see whether vaccinating the mother against Respiratory Syncytial Virus (RSV) increases her baby's defences against the virus. | 32 (10%) | 10 (3.2%) | 270 (87%)* |
| 1. The clinical trial vaccine is a way to protect my baby from illnesses caused by RSV. | 33 (11%) | 14 (4.5%) | 265 (85%)* |
| 1. When I say I want to participate in this clinical trial, I will be put into a group at random (for example, using heads or tails). | 39 (12%) | 29 (9.3%) | 244 (78%)* |
| 1. Throughout the clinical trial, I will know which group I am in. | 35 (11%) | 186 (60%)* | 91 (29%) |
| 1. It has been explained to me how long the clinical trial will last. | 25 (8.0%) | 7 (2.2%) | 280 (90%)* |
| 1. My participation in this clinical trial does not entail any additional risk or discomfort to either my baby or me. | 79 (25%) | 100 (32%)* | 133 (43%) |
| 1. When I am given a placebo it will increase both my defences and those of my baby. | 77 (25%) | 152 (49%)* | 83 (27%) |
| 1. An independent ethics committee has reviewed this clinical trial and approved it. | 62 (20%) | 8 (2.6%) | 242 (78%)* |
| 1. If my baby or I have any health problems from participating in this clinical trial, we can go to my doctor or to the trial doctor. | 24 (7.7%) | 8 (2.6%) | 280 (90%)* |
| 1. After receiving the vaccine, my defences and those of my baby may not have increased. | 83 (27%) | 24 (7.7%) | 205 (66%)* |
| 1. The clinical trial sponsor, its team members or an auditor may have access to my personal information. | 69 (22%) | 56 (18%) | 187 (60%)* |
| 1. The sponsor has taken out a specific insurance policy for this clinical trial. | 111 (36%) | 12 (3.8%) | 189 (61%)* |
| 1. The expenses arising from my participation in the clinical trial will be covered by the clinical trial sponsor. | 47 (15%) | 9 (2.9%) | 256 (82%)* |
| 1. I am clear about who I should talk to if I have any questions or concerns about the clinical trial. | 33 (11%) | 4 (1.3%) | 275 (88%)* |
| 1. By participating in this clinical trial, I am helping researchers gather information that can benefit others. | 15 (4.8%) | 1 (0.3%) | 296 (95%)* |
| 1. I can ask for my blood samples to be destroyed at any time. | 88 (28%) | 24 (7.7%) | 200 (64%)* |
| 1. Participation in this clinical trial is voluntary | 11 (3.5%) | 3 (1.0%) | 298 (96%)* |
| 1. I will have to stay in the clinical trial, even if I want to leave it. | 35 (11%) | 225 (72%)* | 52 (17%) |
| 1. I will be given a copy of the Informed Consent document. | 45 (14%) | 4 (1.3%) | 263 (84%)* |
| 1. I cannot know the results of the clinical trial. | 149 (48%) | 98 (31%)* | 65 (21%) |
| 1. When I sign the Informed Consent for the clinical trial, I know that I agree to participate in it. | 22 (7.1%) | 9 (2.9%) | 281 (90%)* |

**Part B**

|  | Response | | | | |
| --- | --- | --- | --- | --- | --- |
|  | 1 | 2 | 3 | 4 | 5 |
| 1. That the clinical trial vaccine is being researched | 1 (0.3%) | 5 (1.6%) | 21 (6.7%) | 76 (24%) | 209 (67%) |
| 1. That my participation will help future patients. | 2 (0.6%) | 2 (0.6%) | 11 (3.5%) | 48 (15%) | 249 (80%) |
| 1. How long I will be in this clinical trial. | 3 (1.0%) | 6 (1.9%) | 30 (9.6%) | 66 (21%) | 207 (66%) |
| 1. What the researchers want to discover in the clinical trial. | 2 (0.6%) | 3 (1.0%) | 17 (5.4%) | 84 (27%) | 206 (66%) |
| 1. What I will do at each visit. | 6 (1.9%) | 8 (2.6%) | 27 (8.7%) | 72 (23%) | 199 (64%) |
| 1. The possible risks and inconveniences of participating in this clinical trial. | 2 (0.6%) | 6 (1.9%) | 36 (12%) | 106 (34%) | 162 (52%) |
| 1. The possible benefits I can obtain from participating in this clinical trial. | 4 (1.3%) | 6 (1.9%) | 39 (12%) | 97 (31%) | 166 (53%) |
| 1. How my personal data will be protected. | 3 (1.0%) | 12 (3.8%) | 43 (14%) | 88 (28%) | 166 (53%) |
| 1. How my participation may benefit the future development of vaccines that will help other people. | 1 (0.3%) | 3 (1.0%) | 16 (5.1%) | 86 (28%) | 206 (66%) |
| 1. What I should do if my baby or I become ill during the clinical trial. | 2 (0.6%) | 3 (1.0%) | 21 (6.7%) | 94 (30%) | 192 (62%) |
| 1. Who I should talk to if I have any questions or concerns about the clinical trial. | 5 (1.6%) | 3 (1.0%) | 25 (8.0%) | 75 (24%) | 204 (65%) |
| 1. That participation in the clinical trial is not mandatory. | 0 (0%) | 2 (0.6%) | 12 (3.8%) | 43 (14%) | 255 (82%) |
| 1. In general, did you understand the information about the clinical trial well? | 0 (0%) | 1 (0.3%) | 20 (6.4%) | 94 (30%) | 197 (63%) |

### III: Assent Comprehension Questionnaire (C-CAsIn) (adapted from the QuIC) for use in vaccine clinical trials (minors). Parts A and B of each survey and frequency of response (correct answers are identified by a *).

**Part A**

|  | Response | |
| --- | --- | --- |
|  | AGREE | DISAGREE |
| 1. I can decide to participate in this study without discussing it with my parents. Their opinion does not matter. | 130 (21%) | 490 (79%)* |
| 1. One of the benefits of participating in this study is helping other children. What the researchers learn from me can be applied to others. | 596 (96%)* | 24 (3.9%) |
| 1. The researchers have told me how long the study will take. | 585 (94%)* | 35 (5.6%) |
| 1. The study vaccine has been tested before in many girls and boys. | 527 (85%)* | 93 (15%) |
| 1. One of the objectives of this study is to see how safe the product is. | 574 (93%)* | 46 (7.4%) |
| 1. One of the benefits of participating in this study could be improving my defences against diseases. | 587 (95%)* | 33 (5.3%) |
| 1. After I decide to participate in this study, I will be randomly put in a group (like heads or tails). | 565 (91%)* | 55 (8.9%) |
| 1. I will know what group I am put in throughout the whole study. | 279 (45%) | 341 (55%)* |
| 1. If I receive the placebo, my defences will improve. | 236 (38%) | 384 (62%)* |
| 1. Participating in this study does not involve any risk or inconvenience. | 336 (54%) | 284 (46%)* |
| 1. By participating in the study, I would be helping the investigators to know more about the product they study. | 599 (97%)* | 21 (3.4%) |
| 1. The information that I have read explains who I have to talk to if I am worried or if I have any questions. | 592 (95%)* | 28 (4.5%) |
| 1. If I do not want to participate, I can leave the study without any problem. | 589 (95%)* | 31 (5.0%) |
| 1. I have to stay in the study even if I want to quit. | 103 (17%) | 517 (83%)* |

**Part B**

|  | Response | | | | |
| --- | --- | --- | --- | --- | --- |
|  | 1 | 2 | 3 | 4 | 5 |
| 1. That the study vaccine is being investigated. | 7 (1.1%) | 7 (1.1%) | 75 (12%) | 229 (37%) | 302 (49%) |
| 1. That my participation in the study will help other children. | 3 (0.5%) | 4 (0.6%) | 38 (6.1%) | 99 (16%) | 476 (77%) |
| 1. How long will I be in the study. | 7 (1.1%) | 16 (2.6%) | 74 (12%) | 162 (26%) | 361 (58%) |
| 1. What the researchers are trying to achieve by doing this study. | 5 (0.8%) | 7 (1.1%) | 66 (11%) | 187 (30%) | 355 (57%) |
| 1. What will be done at each visit. | 4 (0.6%) | 13 (2.1%) | 52 (8.4%) | 160 (26%) | 391 (63%) |
| 1. The possible risks and inconveniences of participating in this study. | 17 (2.7%) | 22 (3.5%) | 89 (14%) | 209 (34%) | 283 (46%) |
| 1. The possible benefits of participating in the study. | 4 (0.6%) | 11 (1.8%) | 57 (9.2%) | 215 (35%) | 333 (54%) |
| 1. Which people will know that I am participating in the study. | 23 (3.7%) | 29 (4.7%) | 107 (17%) | 184 (30%) | 277 (45%) |
| 1. Whom I will need to talk to if I have any questions or worries about the study. | 3 (0.5%) | 6 (1.0%) | 48 (7.7%) | 162 (26%) | 401 (65%) |
| 1. That it is not compulsory for me to participate in this study. | 4 (0.6%) | 4 (0.6%) | 30 (4.8%) | 121 (20%) | 461 (74%) |

### IV. Questions by domain:

| Domain | Pregnant | Adults |
| --- | --- | --- |
| Nature/purposes of research | A1; A2; A4; A5; A6 | A1; A2; A5; A6; A9 |
| Risks and benefits | A7; A8; A11; A16 | A7; A10; A16 |
| Alternative procedures | A3; | A4; A8 |
| Legal, subject protection | A9; A12; A17 | A3; A12; A17; |
| Compensation | A13; A14 | A13; A14 |
| Contacts; information | A10; A15; A21 | A11; A15; A21 |
| Subject’s rights | A18; A19; A20; A22 | A18; A19; A20; A22 |

| Domain | Minors |
| --- | --- |
| Nature/purposes of research | A3, A5, A7, A8, A9 |
| Risks and benefits | A2, A4, A6, A10, A11 |
| Contacts; information; Subject’s rights* | A1; A12; A13; A14 |

* Contacts, information and subject’s rights were grouped into one domain, as there was only one question on "contacts; information" (A12), which made it very weak for analysis.

### V. CAWI Informed consent for adults (UK)

E-16608

Good morning/evening, thank you again for participating.

We are currently carrying out research for FISABIO, the Foundation for the Promotion of Health and Biomedical Research in the Valencian Community (Spain).

The research is based on the European i-CONSENT project, which aims to improve the information received by potential participants in clinical trials. We would like to show you a number of materials that are displayed when someone participates in a clinical trial and we would like to know if they are easy to read and understand.

Your participation is of great help and completely VOLUNTARY and does not constitute an invitation to participate in a real clinical trial.

Base: All

**P0 [S] Would you like to take part?**

1. Yes
2. No (Scripter: End of interview)

The aim of this study is to get to know people's opinions about the informational materials provided when carrying out a clinical trial. The aim is to find out whether they are easy to read and understand, whether they are transparent and provide all the information necessary for a person to decide whether or not to participate in a clinical trial with sufficient guarantees. To this end we have created different mock clinical trial scenarios and their respective informed consent materials. Furthermore, this will help us to assess the **Informed Consent Comprehension and Quality Questionnaire (c-CaCI) (adapted from the QuIC) for use in vaccine clinical trials.**

Below, we will show you the informed consent materials for one of these scenarios. We will give you access to a web page where you will see information about your possible participation in a mock clinical trial in different formats (such as text, computer graphics or video).

Reading these documents will take about 35-40 minutes. We will then ask you to answer a short questionnaire (about 12 minutes) that will allow us to see whether everything can be understood well or if there are parts that need to be improved.

**Would you please be so kind as to answer a series of questions to determine whether you meet the basic requirements to take part in this research?**

Base: ALL

**P3 [S] Where do you live?**

| 1 | Tees Valley and Durham | 1 | North East |
| --- | --- | --- | --- |
| 2 | Northumberland and Tyne and Wear | 1 | North East |
| 3 | East Riding and North Lincolnshire | 1 | Yorkshire and The Humber |
| 4 | North Yorkshire | 1 | Yorkshire and The Humber |
| 5 | South Yorkshire | 1 | Yorkshire and The Humber |
| 6 | West Yorkshire | 1 | Yorkshire and The Humber |
| 7 | Cumbria | 2 | North West |
| 8 | Cheshire | 2 | North West |
| 9 | Greater Manchester | 2 | North West |
| 10 | Lancashire | 2 | North West |
| 11 | Merseyside | 2 | North West |
| 12 | Derbyshire and Nottinghamshire | 3 | East Midlands |
| 13 | Leicestershire, Rutland and Northamptonshire | 3 | East Midlands |
| 14 | Lincolnshire | 3 | East Midlands |
| 15 | Herefordshire, Worcestershire and Warwickshire | 3 | West Midlands |
| 16 | Shropshire and Staffordshire | 3 | West Midlands |
| 17 | West Midlands | 3 | West Midlands |
| 18 | East Anglia | 4 | East |
| 19 | Bedfordshire and Hertfordshire | 4 | East |
| 20 | Essex | 4 | East |
| 21 | Inner London | 5 | London |
| 22 | Outer London | 5 | London |
| 23 | Berkshire, Buckinghamshire and Oxfordshire | 6 | South East |
| 24 | Surrey, East and West Sussex | 6 | South East |
| 25 | Hampshire and Isle of White | 6 | South East |
| 26 | Kent | 6 | South East |
| 27 | Gloucestershire, Wiltshire and North Somerset | 7 | South West |
| 28 | Dorset and Somerset | 7 | South West |
| 29 | Cornwall and Isles of Scilly | 7 | South West |
| 30 | Devon | 7 | South West |
| 31 | West Wales and The Valleys | 8 | Wales |
| 32 | East Wales | 8 | Wales |
| 33 | North Eastern Scotland | 9 | Scotland |
| 34 | Eastern Scotland | 9 | Scotland |
| 35 | South Western Scotland | 9 | Scotland |
| 36 | Highlands and Islands | 9 | Scotland |
| 37 | Northern Ireland | 10 | Northern Ireland |

**P4 [S]** Please indicate your gender

## Male

## Female

Base: P4=2

## P5 [S] Are you currently pregnant?

1. Yes (Scripter: Pregnant women quota)
2. No

Base: All

## P6 [Q] Could you tell me your age?

|  |
| --- |

1. From 19 to 38 years old (Scripter: Millennial quota)
2. From 39 to 54 years old (Scripter: Generation X quota)

Scripter & Interviewer: Priority is given to the PREGNANT WOMEN TARGET.

- IF COD 1 IN P5 --> PREGNANT WOMEN TARGET
- IF COD 2 IN P5--> ADULT TARGET.

Scripter: Questions for calculating the Social Class.

**STANDARD SOCIAL CLASS QUESTION**

Q.1 Please indicate to which occupational group the Chief Income Earner in your household

belongs, or which group fits best. This could be you: the Chief Income Earner is the person in your household with the largest income. If the Chief Income Earner is retired and has an occupational pension please answer for their most recent occupation. If the Chief Income Earner is not in paid employment but has been out of work for less than 6 months, please answer for their most recent occupation.

Semi or unskilled manual work (e.g. Manual workers, all apprentices to be skilled trades,

Caretaker, Park keeper, non-HGV driver, shop assistant) (D) 01

Skilled manual worker     (e.g. Skilled Bricklayer, Carpenter, Plumber, Painter, Bus/ Ambulance

Driver, HGV driver, AA patrolman, pub/bar worker, etc.) (C2) 02

Supervisory or clerical/ junior managerial/ professional/administrative (e.g. Office worker,

Student Doctor, Foreman with 25+ employees, salesperson, etc.) (C1) 03

Intermediate managerial/ professional/ administrative (e.g. Newly qualified (under 3 years)

doctor, Solicitor, Board director small organisation, middle manager in large organisation,

principle officer in civil service/local government) (B) 04

Higher managerial/ professional/ administrative (e.g. Established doctor, Solicitor, Board

Director in a large organisation (200+ employees, top level civil servant/public service

employee) (A) 05

Student (C1) 06

Casual worker – not in permanent employment (E) 07

Housewife/ Homemaker (E) 08

Retired and living on state pension (E) 09

Unemployed or not working due to long-term sickness (E) 10

Full-time carer of other household member (E) 11

Other (E) 12

What is your education level?

1. **Secondary School**
2. **High School/Tertiary/Tech. College**
3. **University/Higher Education**
4. **None completed**
5. **Postgraduate Education**
6. **Primary School**
7. **Prefer not to say**

## P7 [S] Have you previously participated in clinical trials?

1. Yes
2. No

And finally, in order to participate, we need you to accept this informed consent to participate in this research:

- NAME OF THE RESPONDENT:
- SURNAME OF THE RESPONDENT:

I agree to participate in the study carried out by GfK Emer for the Foundation for the Promotion of Health and Biomedical Research in the Valencian Community (FISABIO) for the i-CONSENT project.

I declare that:

1. I have received sufficient information about the study, the objectives and my participation in it.
2. My participation in this study is completely voluntary.
3. My participation consists solely of viewing different materials, completing a questionnaire and giving my opinion.
4. During my participation, no photographs, videos or audio recordings will be taken of me, nor may I take pictures of the content of the study.
5. I have read and understood all the points of this informed consent and agree to participate.

| Name: **Improvement to guidelines for informed consent, including vulnerable populations from a gender perspective (i-CONSENT)** | | |
| --- | --- | --- |
| Project Summary: The European i-CONSENT project aims to improve the information received by potential participants in clinical trials. Within the framework of the project, a series of recommendations are developed to improve the Informed Consent process, including the vulnerable population and from a gender perspective, with the help of new technologies | | |
| Protocol Nº: 01.00 | Date: 25/11/2019 | Version 01 |
| Sponsor: Javier Díez- Domingo | Researcher: Javier Díez - Domingo | Language: English |

**I AGREE TO PARTICIPATE**
