# Supplementary material for: Digital Informed Consent/Assent in Clinical Trials Among Pregnant Women, Minors, and Adults: Multicountry Cross-Sectional Evaluation of Comprehension and Satisfaction
Source: JMIR Hum Factors. 2025 Aug 15;12:e65569. doi: 10.2196/65569 (PMC12356628; doi:10.2196/65569)
Supplement: Checklist 1 [file humanfactors-v12-e65569-s002.docx]

### VI. The Checklist for Reporting Results of Internet E-Surveys (CHERRIES)

Below is the completed CHERRIES Checklist for the article "Digital Informed Consent/Assent in Clinical Trials: A Multicountry Evaluation of Comprehension and Satisfaction Across Three Vulnerable Populations (Pregnant Women, Minors, and Adults)" by Jaime Fons-Martinez et al.

**DESIGN**

**Survey design:**

The study targeted minors, pregnant women, and adults from three countries: Spain, the United Kingdom, and Romania. The sample was primarily of convenience, recruited through market research company panels, except for minors in Spain, where partial school-based recruitment was used.

**IRB approval and informed consent process:**

The study was approved by the Research Ethics Committee of the Foundation for the Promotion of Health and Biomedical Research of the Valencian Community (FISABIO) (Approval No. 20200109/09).

**Informed consent:**

All participants provided informed consent prior to participation. For minors, parental or guardian consent was obtained in addition to the child’s assent. Online participants provided consent or assent electronically via a digital form integrated into the survey platform.

**Data protection:**

All data collected were anonymised and securely stored on password-protected servers. Personal identifiers were removed prior to analysis, and participants were assured that their responses would remain confidential.

**DEVELOPMENT AND PRE-TESTING**

Materials were prepared in accordance with the i-CONSENT guidelines, considering the preferences and needs of potential participants. Participatory methods were used for co-design, including design thinking sessions with minors and pregnant women, and online surveys with adults.

The survey was developed using adapted versions of the Quality of Informed Consent questionnaire (QuIC), tailored to each target population and regulatory requirements. Pregnant women and minors participated in pre-testing the questionnaire, providing feedback that informed the final version. The survey was originally drafted in Spanish and professionally translated into English and Romanian.

Due to the situation generated by the COVID-19 pandemic, a pilot study was conducted in Spain in July 2020 to assess the willingness of the target population to respond to the survey depending on the modality (face-to-face, at a GfK-designated location, or at home). The results are as follows:

|  | **TOTAL** | **GENERATION X** | **MILLENNIALS** | **PREGNANT TARGET GROUP** |
| --- | --- | --- | --- | --- |
| 1. Agreed to participate immediately at the time of recruitment (face-to-face) | 0 | 0 | 0 | 0 |
| 2. Provided details for participating in the research from the offices set up by GfK. | 5 | 5 | 0 | 0 |
| 3. Provided email address for participating in the research from home | 58 | 32 | 24 | 2 |
| 4. Declined to participate in the study | 467 | 241 | 211 | 15 |
| **Total individuals contacted** | 530 | 278 | 235 | 17 |

**Testing usability and technical functionality:**

Representatives from the target populations (minors and pregnant women) tested the adapted questionnaires during pre-testing sessions to ensure usability and relevance.

**RECRUITMENT PROCESS**

**Open versus closed survey:**

Participants accessed the materials and survey via a private link sent to their email or provided during recruitment.

**Contact mode:**

Initial contact with potential participants was primarily conducted through the market research company’s panel. For minors in Spain, part of the recruitment (187 out of 312 students) was conducted in schools.

**Survey advertisement:**

Recruitment was carried out through a market research company with established panel databases (30,000 panellists in Spain, 700,000 in Romania, and 2 million in the United Kingdom). In Spain, due to the smaller panel size for pregnant women, part of the recruitment was conducted face-to-face near health centres in Valencia and Madrid by professional recruiters, and for minors, in schools.

**SURVEY ADMINISTRATION**

**Context:**
Materials were accessed via a dedicated website. On the website, participants could choose their preferred format for receiving the consent information, including a layered web page, a narrative or question-and-answer video, or a document with the same text and images as the web format, which could be printed if they preferred to read on paper.

**Mandatory/voluntary participation:**

Participation in the study was voluntary.

**Incentives:**
Participants received compensation according to their recruitment group:

- Minors in Spain: Schools where surveys were conducted received an Amazon voucher worth €200 as an institutional incentive.
- Adults and Pregnant Women: Participants recruited via the market research company’s panel database received points equivalent to €4–5, redeemable for gift cards or other rewards through the panel’s system.

**Date/Time:**
Fieldwork was conducted between October and November 2020.

**Randomisation of items or questionnaires:**

Not applicable.

**Adaptive questionnaire:**

Not applicable.

**Number of items:**

For adults and pregnant women, Part A of the questionnaire comprised 22 questions and Part B comprised 13 questions. For minors, Part A comprised 14 questions and Part B comprised 10 questions.

**Number of survey screens:**

Between 25 and 30 screens.

**Completeness check:**

Upon completion of the survey, the *Confirmit* variable “status=complete” indicated that the interview had been successfully completed and stored on the server. The survey did not allow progression unless all response options were completed.

**Review step:**

Respondents could not move backwards or forwards in the survey once it had been completed. When reaching the section with the consent information, a second browser tab was opened, allowing participants to complete the survey while keeping the information available for reference.

**RESPONSE RATES**

**Unique site visitor:**

In *Confirmit*, each link is unique and individual; once completed, it cannot be reused by another participant. GfK employs *Gatekeeper*, an internally developed real-time de-duplication application (“*digital fingerprinting*”) to ensure that only one survey is completed per participant.

**View rate:**

Page view data were not collected; instead, the status of contacted individuals was recorded as one of three categories: “*completed*”, “*quota already filled*”, or “*excluded for not meeting eligibility criteria*”.

**Participation rate:**

| **Status** | **Spain Adults** | **Spain Pre-adolescents** | **Romania Adults** | **Romania Pre-adolescents** | **UK Adults** | **UK Pre-adolescents** |
| --- | --- | --- | --- | --- | --- | --- |
| **Completed** | 584 | 312 | 222 | 125 | 336 | 183 |
| **Quota already filled** | 906 | 102 | 954 |  | 2,664 |  |
| **Excluded for not meeting eligibility criteria** | 36 | 1,300 | 216 | 423 | 1,334 | 1,695 |

**Completion rate:**

| **Spain Adults** | **Spain Pre-adolescents** | **Romania Adults** | **Romania Pre-adolescents** | **UK Adults** | **UK Pre-adolescents** |
| --- | --- | --- | --- | --- | --- |
| 38% | 18% | 16% | 23% | 8% | 10% |

**PREVENTION OF MULTIPLE ENTRIES:**

**Cookies used and IP check:**

Gatekeeper (includes digital fingerprint and IP address checks)

All three countries had Gatekeeper enabled, with verification set to "Use fingerprint and IP address." This is an Atlas configuration, which checks the respondent’s ID before allowing access to the survey.

Currently, there are two available settings in Atlas for discarding duplicates:

- Directly at the time of Gatekeeper verification, meaning that if a respondent fails the Gatekeeper check, no record is created in the database.
- Within the survey itself, meaning that respondents enter the survey, create a record in the database, but are immediately excluded without proceeding through the survey.

About Gatekeeper: Gatekeeper is GfK’s internally developed real-time de-duplication application. It enables the identification of the browsers used by respondents to complete surveys, in order to detect duplicate respondents.

A respondent completing a survey for the first time is tagged with a unique identifier (GUID) based on the browser used to access the survey. This unique ID is stored in the browser’s local storage (not in cookies). If the respondent attempts to complete the same survey from the same computer, they can easily be identified as a duplicate. Even if the respondent tries to complete the survey from the same browser using a different respondent ID, the system will recognise that the same person is attempting to participate again.

For IP addresses, the same logic is applied as with fingerprinting, except that, instead of duplicating based on the browser’s unique key, the IP address is used.

Local storage does not involve the use of cookies. Local storage is a type of storage available only on the client side (browser); it is not automatically sent to the server and is persistent (it remains stored even after closing the browser, unless the user deletes it manually). Due to its persistence and separation from server requests, local storage is more effective than cookies for identifying duplicate respondents in surveys.

**Log file analysis:**

- Log file retention:

Log files are retained for a limited period for each survey, but they are never analysed by default. They are only examined if issues are reported. In this study the survey was archived during 5 years.

- Record Keeping

All three studies used a sample table with unique identifiers created for each respondent, in the following format: 0042B0DD-BB94-48BA-A1CA-D2396F893914

This means that there is no way for a respondent to have accessed the survey via open links.

This approach significantly reduces the risk of duplicates in the survey database. Even if a respondent had somehow gained access to multiple links, they would have been excluded by Gatekeeper (assuming the same browser/IP was used).

**ANALYSIS:**

**Handling of Incomplete Questionnaires**

Six individuals whose response patterns included more than 80% “*Don’t know*” responses in Part A were excluded from the analyses.

**Questionnaires with atypical timestamps:**

Surveys with a duration 33% above or below the average were excluded.

**Statistical correction:**

Linear regression models were used and an ordered quantile normalisation transformation was applied as the mean score did not follow a normal distribution. Interactions between variables were also tested, using the AIC (Akkaike information criterion) value to penalise model fit according to the number of variables used.
